# Retraction Note: Effects of replacing groundnut cake with rumen content supplemented with or without enzyme in the diet of weaner rabbits

**DOI:** 10.1186/s12944-016-0265-9

**Published:** 2016-05-27

**Authors:** A. A. Adeniji, S. Rumak, R. A. Oluwafemi

**Affiliations:** Department of Animal Science, Faculty of Agriculture, University of Abuja, P.M.B. 117 FCT Abuja, Nigeria

This article [1] has been retracted by the publisher because after submission to the *Journal of Animal Science and Biotechnology*, it was published in *Lipids in Health and Disease* due to a human error. The authors have not responded to any of our communications. We sincerely apologise to all parties concerned.
